# Delayed High-Output Chylothorax Following Thymectomy for Thymoma

**DOI:** 10.7759/cureus.119153

**Published:** 2026-11-05

**Authors:** Mourad Nafaa, Radia Bencherki, Jihane Ziati, Mustapha Noussair, Belyamani Lahcen

**Affiliations:** 1 Emergency Medicine, Cheikh Khalifa International University Hospital/Faculty of Medicine, Mohammed VI University of Health and Sciences, Casablanca, MAR; 2 Clinical Pharmacy, Cheikh Khalifa International University Hospital, Casablanca, MAR; 3 Anesthesia and Critical Care, Cheikh Khalifa International University Hospital/Faculty of Medicine, Mohammed VI University of Health and Sciences, Casablanca, MAR; 4 Anesthesiology and Intensive Care Medicine, Mohammed V Military Hospital, Rabat, MAR

**Keywords:** chylothorax, conservative management, delayed chylothorax, high-output chylothorax, myasthenia gravis, octreotide, thymectomy, thymoma, vats

## Abstract

Chylothorax is an uncommon complication following thymectomy and results from disruption of the thoracic duct or its tributaries during surgery. We report the case of a 50-year-old man with autoimmune myasthenia gravis who developed a delayed postoperative chylothorax following video-assisted thoracoscopic surgery (VATS) thymectomy for a type B2 thymoma, Masaoka stage I. On postoperative day 21, the patient presented with acute respiratory distress and severe hypoxemia. Chest computed tomography revealed a large left-sided pleural effusion with extensive passive lung collapse. Thoracentesis in the emergency department evacuated 1.5 L of milky pleural fluid, followed by an additional 4 L after chest tube drainage in the intensive care unit. Pleural fluid analysis revealed a triglyceride concentration of 188 mg/dL, with chylomicrons detected, confirming the diagnosis of chylothorax. The fluid also showed marked lymphocytic predominance (96%). Management included pleural drainage, a strict fat-free diet, and octreotide, administered as a 100 µg slow intravenous loading dose followed by continuous infusion at 50 µg/h. Pleural drainage showed an overall decreasing trend and eventually ceased. Octreotide was discontinued after 13 calendar days, and the chest tube was removed without requiring additional invasive or surgical intervention. The patient was subsequently discharged home. This case illustrates that post-thymectomy chylothorax may present several weeks after surgery and that conservative management may achieve a favorable outcome even in the setting of substantial initial pleural drainage.

## Introduction

Chylothorax is characterized by the accumulation of chyle within the pleural cavity following disruption of the thoracic lymphatic system. Chyle is a lymphatic fluid originating from the intestine and contains lipids, proteins, electrolytes, and predominantly lymphocytes. Persistent chyle loss may therefore result in important nutritional, metabolic, and immunological consequences [1].

Postoperative chylothorax may result from iatrogenic injury to the thoracic duct or its tributaries during thoracic surgery. The clinical picture varies according to how much chyle accumulates and how quickly it does so, while persistent leakage may lead to significant nutritional and metabolic consequences [2].

Although postoperative chylothorax is a recognized complication of thoracic surgery, its occurrence following thymectomy remains uncommon. Huang et al. reported three cases among 217 patients undergoing extended thymectomy for myasthenia gravis, corresponding to an incidence of 1.38%. While this complication is generally recognized during the early postoperative period, delayed presentations may also occur [3].

Management depends on the severity and persistence of the lymphatic leak. Conservative approaches, including dietary fat restriction, pleural drainage, and pharmacological treatment such as octreotide, aim to reduce chyle flow and may promote spontaneous resolution in selected patients, whereas persistent high-output leaks may require invasive intervention [4]. 

We report the case of a 50-year-old man who developed a large left-sided chylothorax presenting with acute respiratory distress 21 days after video-assisted thoracoscopic thymectomy for a type B2 thymoma, Masaoka stage I. This case highlights an unusually delayed presentation of this rare postoperative complication and discusses its diagnostic and therapeutic management in comparison with previously reported cases.

## Case presentation

A 50-year-old man was admitted to the intensive care unit on postoperative day 21 following video-assisted thoracoscopic surgery (VATS) thymectomy for a type B2 thymoma, Masaoka stage I [5], with acute respiratory distress secondary to postoperative chylothorax.

His symptoms had started the day before presentation to the emergency department, with the sudden onset of severe dyspnea at rest, without other associated symptoms. He remained afebrile but reported marked asthenia.

His medical history was notable for recently diagnosed autoimmune myasthenia gravis, treated with ambenonium, prednisone, and azathioprine. He had undergone VATS thymectomy for a thymoma, with histopathological and immunohistochemical examination revealing a type B2 thymoma, Masaoka stage I. The postoperative course was complicated by a myasthenic crisis, followed by the development of chylothorax secondary to thoracic surgery.

Emergency department assessment and initial management

On admission to the emergency department, the patient was conscious, with a Glasgow Coma Scale score of 15/15. He was tachypneic and hypoxemic, with an oxygen saturation (SpO₂) of 73%, which improved to 97% with oxygen delivered through a non-rebreather mask. Pulmonary examination revealed rhonchi over the left lung base. Cardiovascular examination revealed normal S1 and S2 heart sounds with tachycardia and no additional murmurs. There was no calf tenderness or lower-limb edema. The abdomen was soft and non-tender. Profuse sweating was also noted.

Initial management in the emergency department included oxygen therapy via a non-rebreather mask, peripheral intravenous access, monitoring, intravenous fluid administration, and placement in a semi-seated position. Initial laboratory investigations were subsequently performed, with the main findings summarized in Table 1.

**Table 1:** Laboratory findings on admission to the emergency department.

Laboratory parameter	Value	Normal range
Complete blood count		
Hemoglobin	16.8 g/dl	13 - 18 g/dL
White blood cells	18.11×10³/mm³	4 - 11×10³/mm³
Neutrophils	16.35×10³/mm³	1.4 - 7.7 ×10³/mm³
Lymphocytes	0.83×10³/mm³	1.0 - 4.8 ×10³/mm³
Platelets	279×10³/mm³	150 - 400 ×10³/mm³
Electrolytes		
Sodium (Na⁺)	139 mEq/l	136 - 145 mEq/l
Potassium (K⁺)	4.1 mEq/l	3.5 - 5.1 mEq/l
Chloride (Cl⁻)	102 mEq/l	98 - 107 mEq/l
Calcium (Ca²⁺)	85 mg/L	90 - 102 mg/L
Bicarbonate	27 mEq/l	22 - 31 mEq/l
Total protein	76 g/L	64 - 83 g/L
Inflammatory markers		
C-reactive protein (CRP)	2.80 mg/L	< 5 mg/L
Coagulation assessment		
D-dimer	9.03 mg/L	< 0.50 mg/L
Cardiac biomarkers		
Troponin	0.154 ng/ml	< 0.034
NT-proBNP	228.8 pg/mL	< 125 (non-acute setting); < 300 (acute setting)
Liver function tests		
AST (aspartate aminotransferase)	28 UI/L	11 - 34 UI/L
ALT (alanine aminotransferase)	40 UI/L	< 45 UI/L
Renal function		
Urea	0.40 g/L	0.18 - 0.55 g/L
Creatinine	8.80 mg/L	6.0 - 13.0 mg/L

A chest computed tomography (CT) scan was subsequently performed, revealing a large left-sided pleural effusion with extensive passive lung collapse. The radiological report recommended correlation with the other clinical and diagnostic findings (Figure 1).

**Figure 1:**
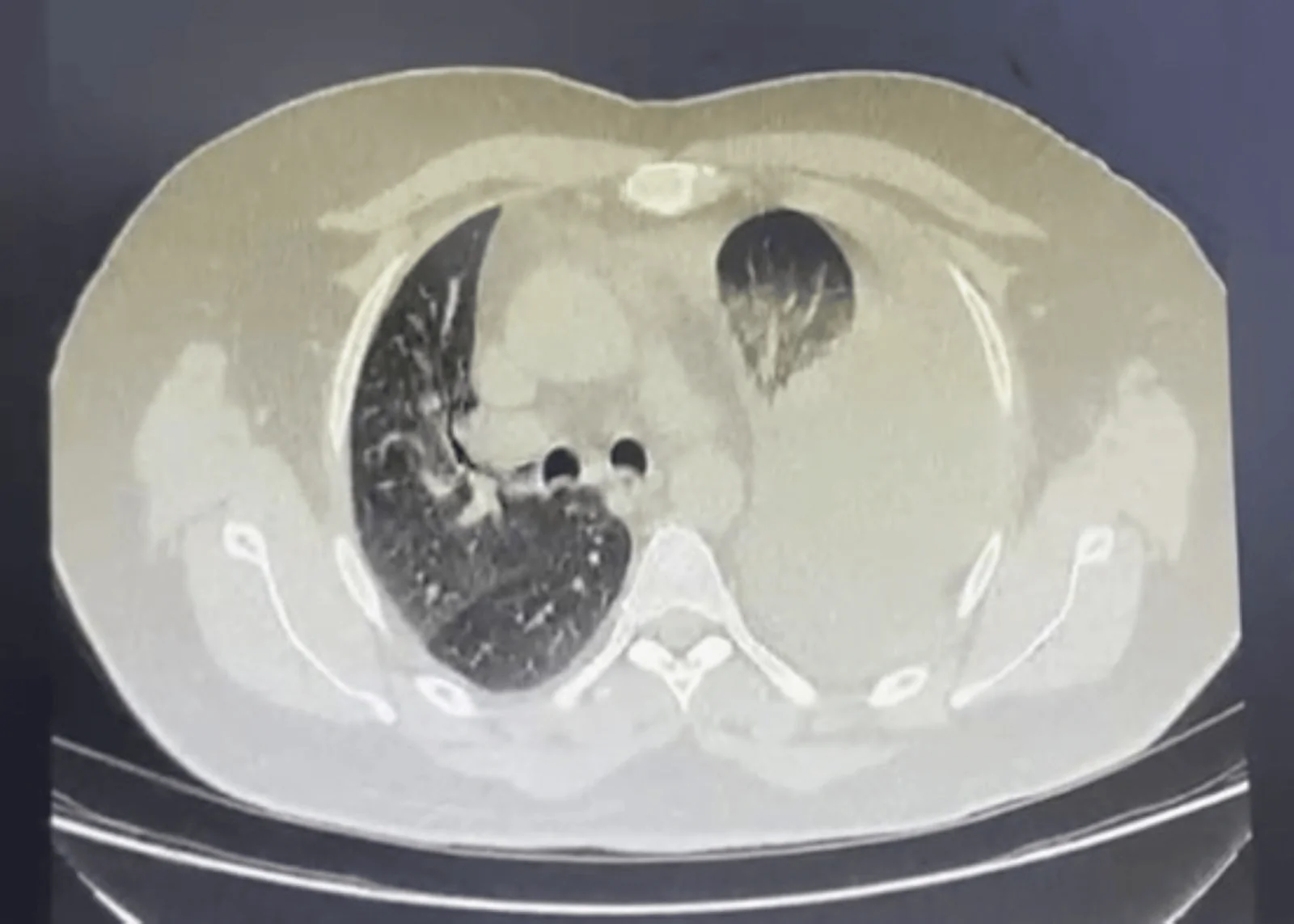
Chest computed tomography scan showing a large left-sided pleural effusion with extensive passive collapse of the left lung.

Given the large volume of the pleural effusion and its respiratory impact, pleural drainage was performed in the emergency department, resulting in the evacuation of 1.5 L of pleural fluid. A sample was sent to the laboratory for biochemical, cytological, and microbiological analyses. Pleural fluid analysis revealed a triglyceride concentration of 188 mg/dL, with chylomicrons detected, confirming the diagnosis of chylothorax. The fluid was also characterized by a lymphocyte predominance (96%). The complete results are presented in Table 2.

**Table 2:** Results of pleural fluid analysis following drainage in the emergency department.

Parameter	Emergency department sample
Volume drained	1.5 L
Macroscopic appearance	Milky
Protein	41.04 g/L
Glucose	1,27 g/L
Triglycerides	188 mg/dL
Chylomicrons	Present
Lactate dehydrogenase (LDH)	155 UI/L
White blood cells	28 800/mm³
Red blood cells	980/mm³
Neutrophils	4%
Lymphocytes	96%
Fungal elements	Absent
Visible microorganisms	Absent
Culture	Negative

After admission to the intensive care unit and further pleural drainage, oxygen saturation reached 99%. Although the patient's postoperative course had previously been complicated by a myasthenic crisis, no clinical signs of an ongoing crisis were observed at ICU admission.

A cardiological evaluation was subsequently performed as part of the assessment of dyspnea. Electrocardiography showed a regular sinus rhythm at 110 beats/min, with no repolarization abnormalities. Transthoracic echocardiography revealed preserved biventricular function, with a left ventricular ejection fraction of 60%, no significant mitral or aortic valvular disease, no elevation of left ventricular filling pressures, and no pulmonary hypertension. No pericardial effusion was observed. A moderate-to-large left-sided pleural effusion was also noted.

According to the cardiology assessment, the elevated D-dimer and troponin levels could be related to hypoxemia. Given the clinical context, ventilation/perfusion lung scintigraphy was proposed to investigate the possibility of pulmonary embolism if respiratory status failed to improve following pleural drainage. CT pulmonary angiography was also considered but was not performed following cardiology consultation. The Geneva score was 2, indicating a low clinical probability of pulmonary embolism.

Intensive care unit management

Given the persistence of the pleural effusion and respiratory distress, the patient was transferred to the intensive care unit (ICU). On ICU admission, he was conscious, with a Glasgow Coma Scale score of 15/15, and hemodynamically stable, with a blood pressure of 120/60 mmHg and a heart rate of 87 beats/min. Oxygen saturation was 96% while receiving oxygen through a non-rebreather mask. Pulmonary examination revealed decreased breath sounds over the left lung field. Cardiovascular examination revealed normal S1 and S2 heart sounds with no additional sounds. The abdomen was soft. There was no calf tenderness or lower-limb edema. The patient was afebrile, with a temperature of 37°C.

Additional chest tube drainage was performed, resulting in the evacuation of 4 L of pleural fluid (Figure 2). The drained fluid had a milky appearance, consistent with chylothorax in this postoperative setting. A second pleural fluid sample was sent to the laboratory for biochemical, cytological, and microbiological analyses.

**Figure 2:**
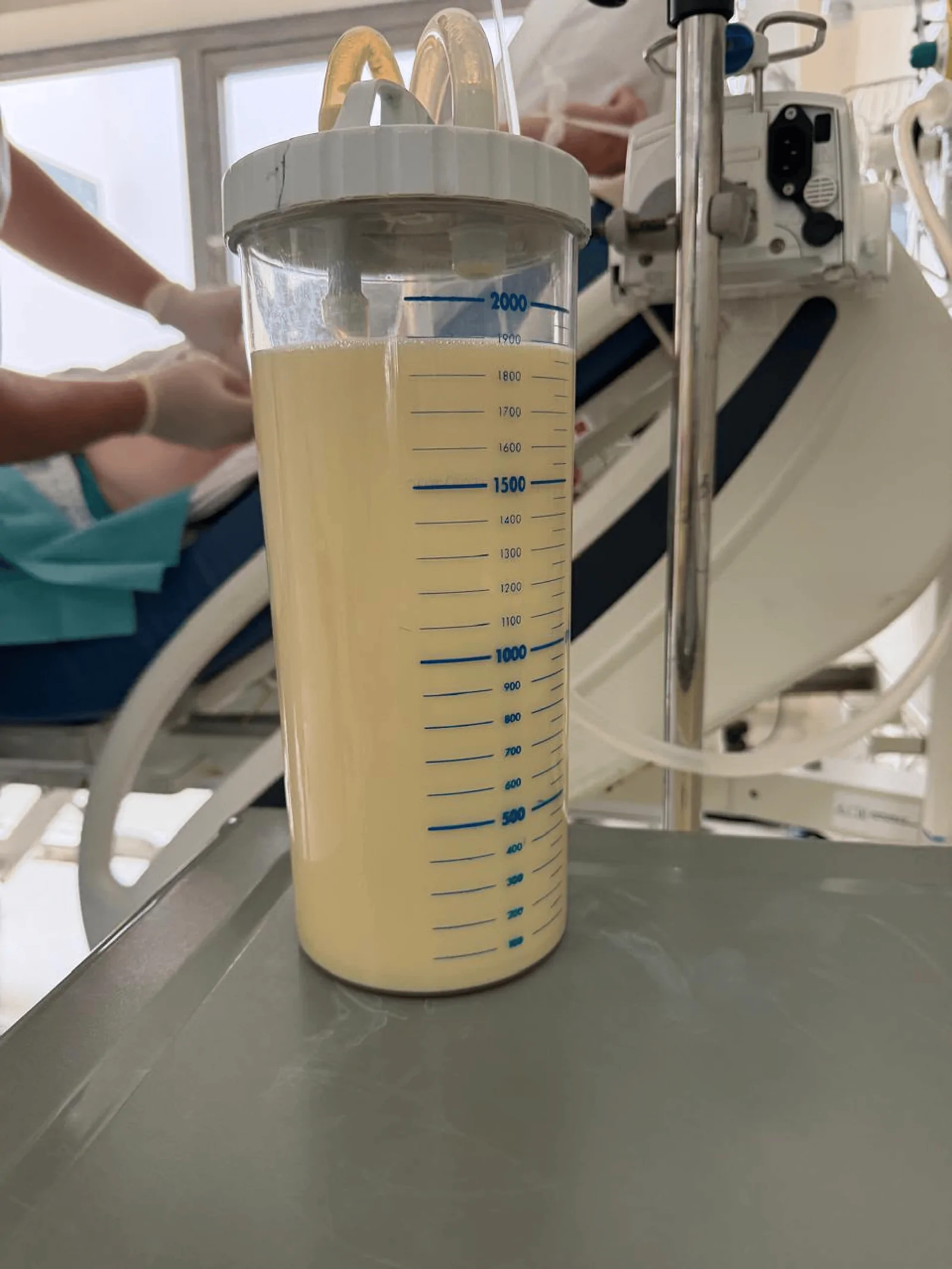
Pleural drainage on the first day of ICU admission.

Pleural fluid analysis revealed a protein concentration of 33.53 g/L. Cytological analysis showed a few leukocytes, with no fungal elements or visible microorganisms. Bacterial culture was negative. The complete results of the pleural fluid analysis performed in the ICU are presented in Table 3.

**Table 3:** Results of pleural fluid analysis following chest tube drainage in the ICU.

Parameter	ICU sample
Volume drained	4 L
Macroscopic appearance	Milky
Protein	33.53 g/L
Glucose	1.12 g/L
Lactate dehydrogenase (LDH)	188 UI/L
White blood cells	Few
Red blood cells	Numerous
Fungal elements	Absent
Visible microorganisms	Absent
Culture	Negative

Following chest tube drainage, repeat laboratory investigations were performed. The complete blood count showed a decrease in hemoglobin from 16.8 to 14.3 g/dL, along with a decrease in white blood cell count from 18.11 to 9.77 × 10³/mm³ and neutrophil count from 16.35 to 5.55 × 10³/mm³. The lymphocyte count increased from 0.83 to 3.20 × 10³/mm³, while the platelet count decreased from 279 to 143 × 10³/mm³.

Regarding fluid and electrolyte parameters, serum sodium decreased from 139 to 134 mEq/L, while potassium remained overall stable (4.1 to 4.2 mEq/L) and chloride remained unchanged at 102 mEq/L. Bicarbonate decreased from 27 to 19 mEq/L. Total protein decreased from 76 to 64 g/L, and serum calcium from 85 to 80 mg/L.

Regarding inflammatory markers, C-reactive protein (CRP) increased from 2.80 to 14.70 mg/L, while procalcitonin (PCT) was 0.02 ng/mL on repeat testing. Regarding renal function, urea decreased from 0.40 to 0.23 g/L, while serum creatinine decreased from 8.80 to 7.70 mg/L. Changes in the laboratory parameters following drainage are presented in Table 4.

**Table 4:** Laboratory findings after chest tube drainage in the ICU.

Laboratory parameter	Value	Normal range
Complete blood count		
Hemoglobin	14.3 g/dl	13 - 18 g/dL
White blood cells	9.77×10³/mm³	4 - 11×10³/mm³
Neutrophils	5.55×10³/mm³	1.4 - 7.7 ×10³/mm³
Lymphocytes	3.20×10³/mm³	1.0 - 4.8 ×10³/mm³
Platelets	143×10³/mm³	150 - 400 ×10³/mm³
Electrolytes		
Sodium (Na⁺)	134 mEq/l	136 - 145 mEq/l
Potassium (K⁺)	4.2 mEq/l	3.5 - 5.1 mEq/l
Chloride (Cl⁻)	102 mEq/l	98 - 107 mEq/l
Calcium (Ca²⁺)	80 mg/L	90 - 102 mg/L
Bicarbonate	19 mEq/l	22 - 31 mEq/l
Total protein	64 g/L	64 - 83 g/L
Inflammatory markers		
C-reactive protein (CRP)	14.70 mg/L	< 5 mg/L
Infectious marker		
Procalcitonin (PCT)	0.02 ng/mL	< 0.50 ng/mL
Renal function		
Urea	0.23 g/L	0.18 - 0.55 g/L
Creatinine	7.70 mg/L	6.0 - 13.0 mg/L

On day 2 of ICU hospitalization, the chest tube remained productive, with 1,100 mL of pleural fluid collected over 24 hours. The fluid retained a chylous appearance, with a few blood clots. In Figure 3, the drainage system shows a total volume of 1,600 mL; however, 500 mL corresponded to added normal saline, resulting in an actual pleural drainage volume of 1,100 mL.

**Figure 3:**
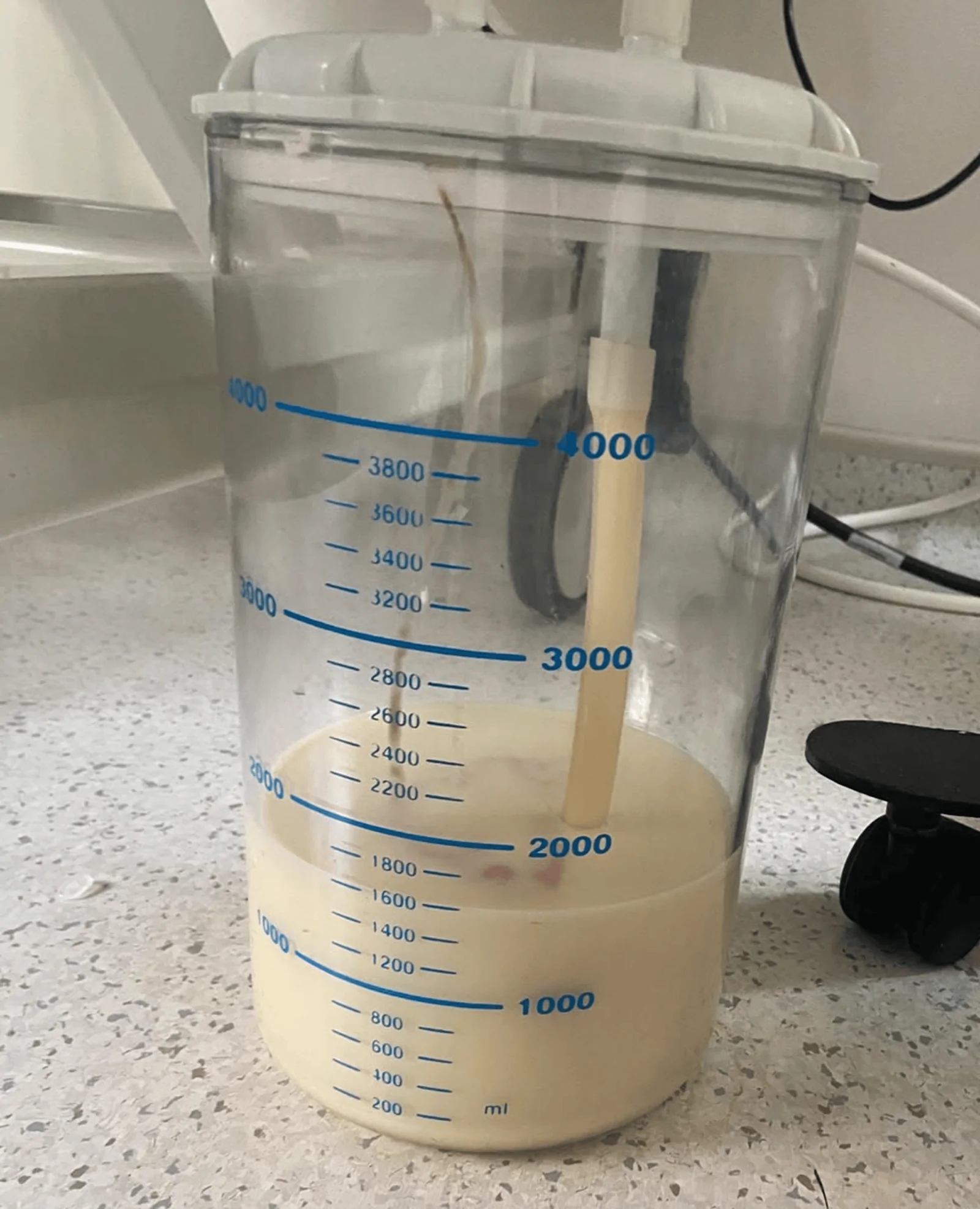
Pleural drainage on day 2 of ICU hospitalization showing chylous-appearing fluid with a few blood clots. The actual pleural drainage volume was 1,100 mL, with the additional 500 mL corresponding to added normal saline.

A follow-up chest radiograph performed on day 3 of the ICU stay, after pleural drainage, showed marked improvement in left lung aeration (Figure 4).

**Figure 4:**
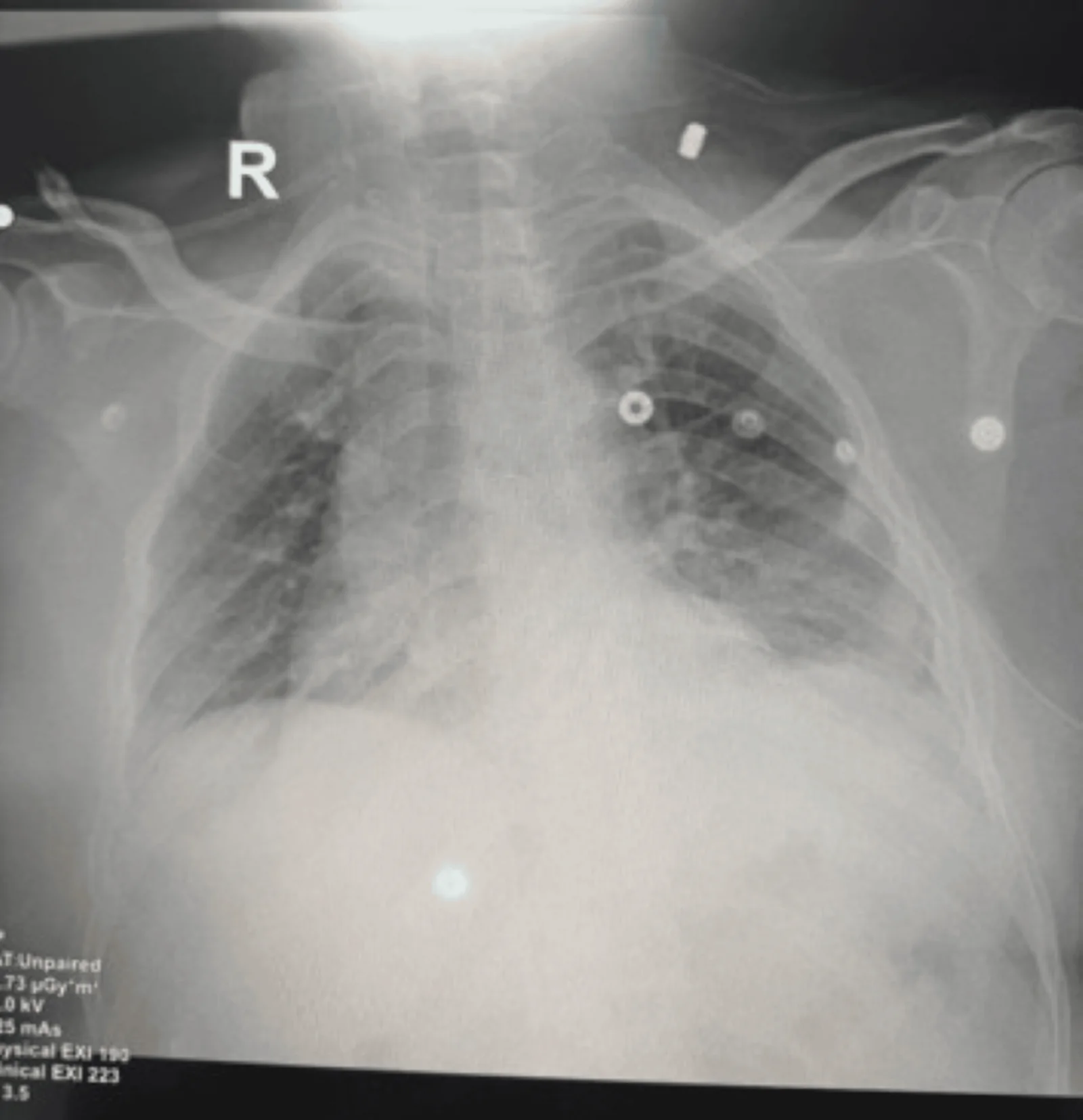
Follow-up chest radiograph showing marked improvement in left lung aeration following drainage of a pleural effusion associated with postoperative chylothorax on ICU day 3.

A thoracic surgery consultation was obtained. The surgical team recommended daily quantification of pleural fluid output to monitor its evolution. Chest tube removal was to be considered once drainage had ceased, in order to allow healing.

Pleural drainage output showed an overall decreasing trend during hospitalization, as illustrated in Figure 5.

**Figure 5:**
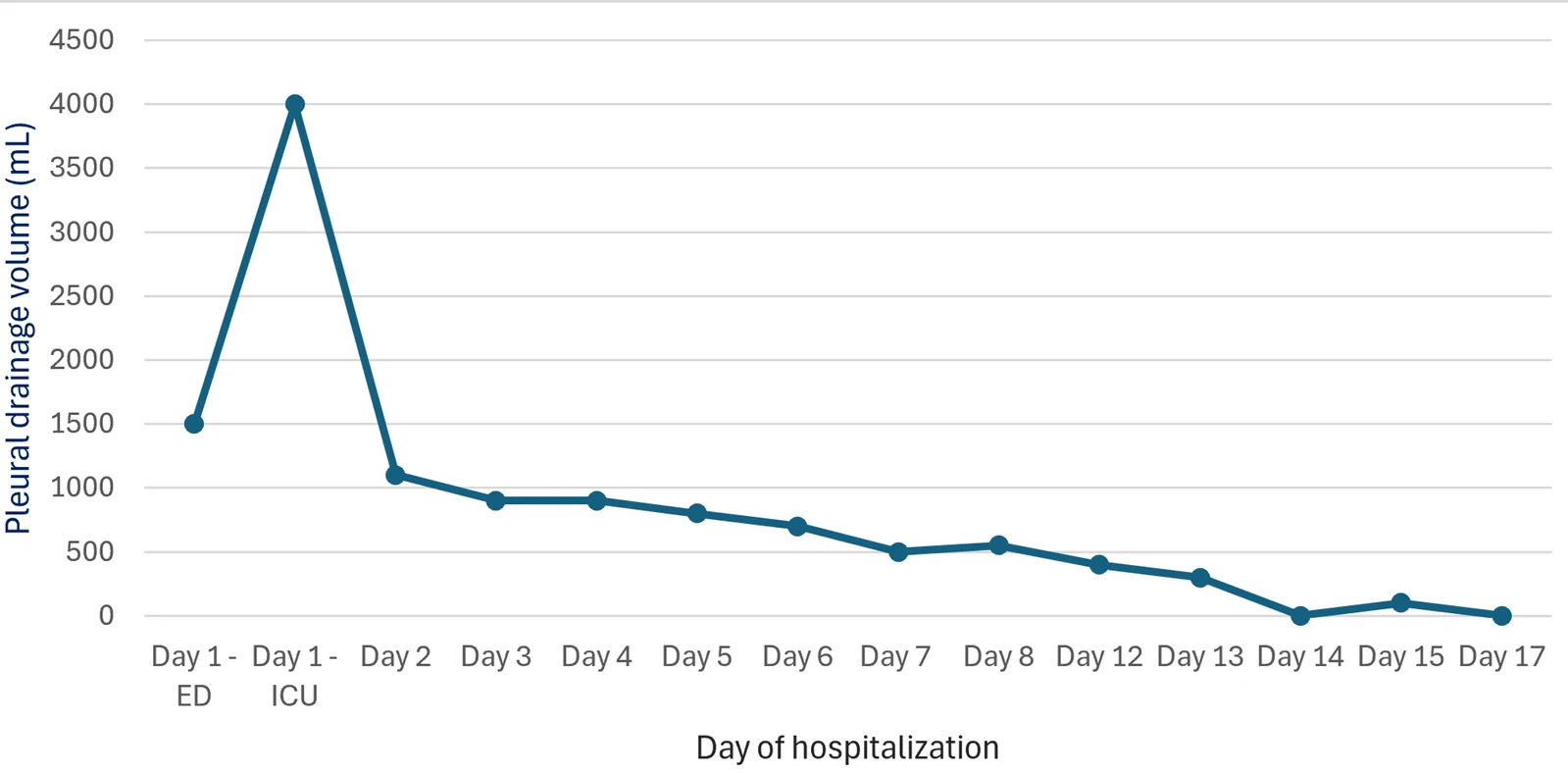
Evolution of pleural drainage volume during hospitalization.

Pleural drainage output was monitored daily throughout hospitalization (Figure 5). In the emergency department, thoracentesis resulted in the evacuation of 1,500 mL of pleural fluid. Following transfer to the ICU, chest tube drainage yielded 4,000 mL on day 1 and remained productive on day 2, with 1,100 mL collected over 24 hours. Drainage output subsequently decreased to 900 mL on days 3 and 4, 800 mL on day 5, 700 mL on day 6, and 500 mL on day 7. On day 8, 550 mL of pleural fluid was collected. Thereafter, documented drainage output was 400 mL on day 12 and 300 mL on day 13.

On day 14, no pleural fluid was collected. A low drainage output resumed on day 15, with 100 mL collected. A follow-up anteroposterior chest radiograph performed on day 15 showed mild bilateral interstitial changes, blunting of the left costophrenic angle, and a preserved cardiomediastinal silhouette (Figure 6).

**Figure 6:**
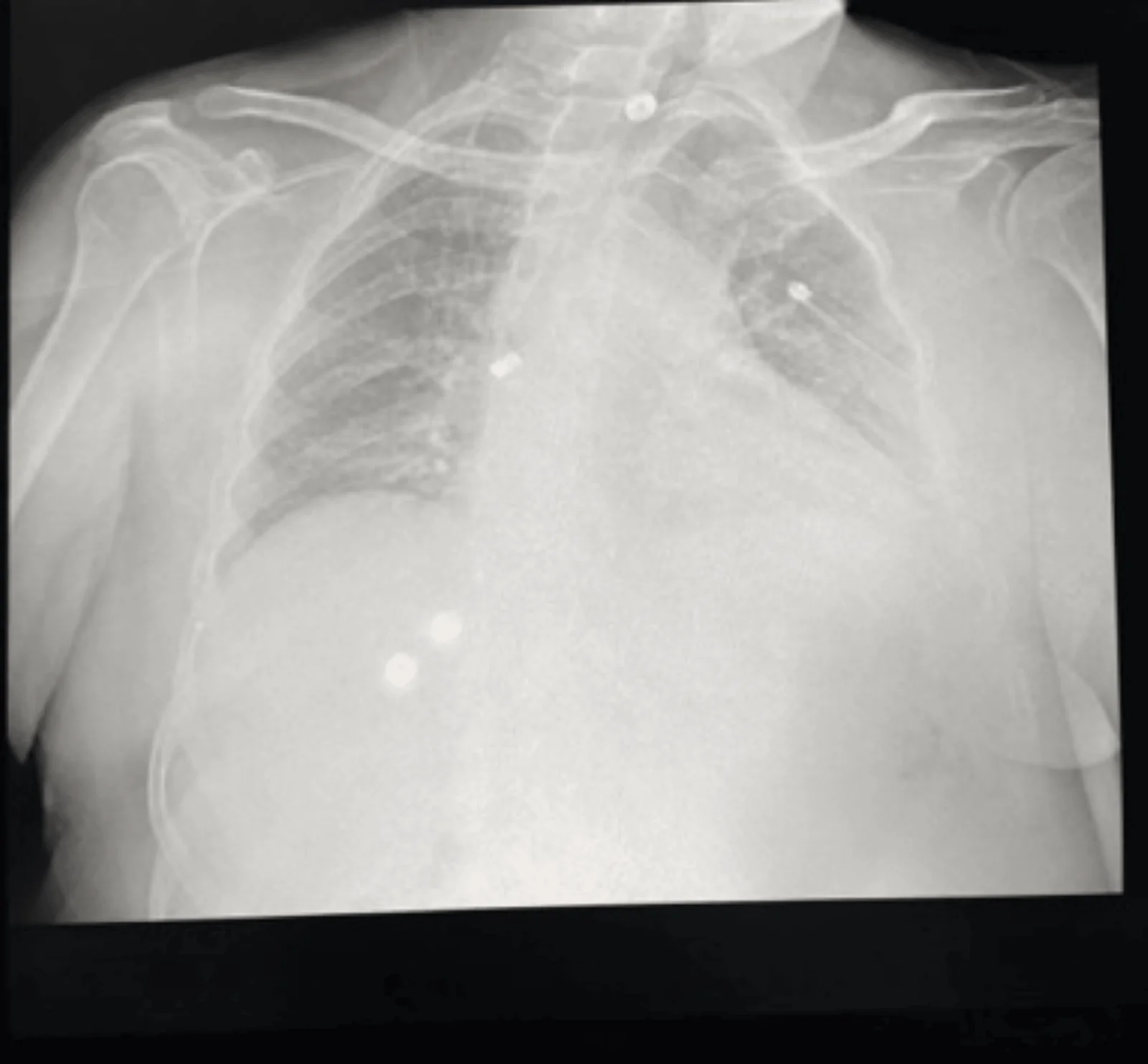
Follow-up anteroposterior chest radiograph showing residual left-sided pleural effusion associated with postoperative chylothorax on ICU day 15.

On day 17, the chest tube no longer yielded any fluid, and by day 18, only traces of serohematic fluid persisted. A further follow-up chest radiograph performed on day 18 showed regression of the pleural effusion with re-expansion of the left lung; the left-sided chest tube remained in place (Figure 7).

**Figure 7:**
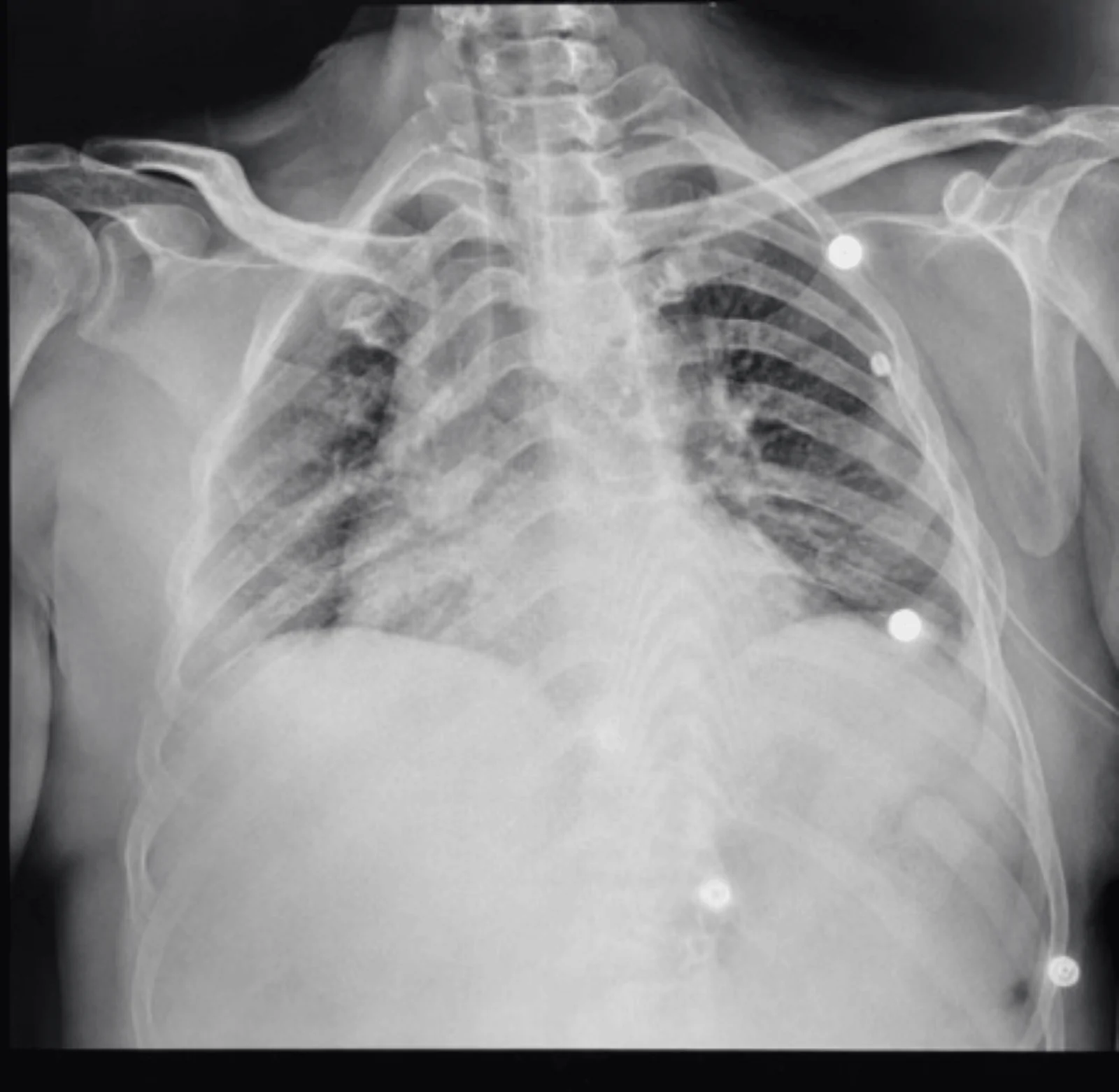
Frontal chest radiograph showing regression of left-sided pleural effusion associated with postoperative chylothorax on ICU day 18.

Therapeutic management in the ICU

On admission to the ICU (day 1), therapeutic management included intravenous normal saline and glucose solutions, with electrolyte supplementation including sodium, potassium, and calcium added to the intravenous fluids. Antibiotic therapy consisted of ceftriaxone 2 g/day, as well as thromboprophylaxis with enoxaparin 0.4 mL/day. The patient also received intravenous omeprazole, his usual treatment for myasthenia gravis, and supplementation with potassium chloride syrup, calcium-vitamin D3, and vitamin C. Normal saline was discontinued on days 2-3, while the remainder of the treatment was maintained.

Following confirmation of the diagnosis of chylothorax, a strict fat-free diet was initiated. On postoperative day 7, octreotide therapy was introduced, starting with an intravenous loading dose of 100 µg, followed by continuous infusion at 50 µg/h using a syringe pump. On postoperative day 8, analgesic treatment with paracetamol (1 g three times daily) was initiated.

Biological evolution

Laboratory monitoring during the ICU stay showed a favorable evolution of the inflammatory response, with CRP decreasing from 14.70 mg/L on the post-drainage assessment to 5.50 mg/L on day 5 and 1.30 mg/L on day 8. CRP remained low on subsequent assessments. Procalcitonin levels also remained low throughout follow-up.

Regarding fluid and electrolyte parameters, serum sodium, initially 134 mEq/L, increased to 139 mEq/L on day 5 and remained overall stable thereafter. Hypokalemia of 3.3 mEq/L was observed on day 5 and persisted on day 8. Serum potassium subsequently increased to 3.5 mEq/L before decreasing to 3.1 mEq/L on day 18; potassium supplementation was maintained.

A progressive decrease in total protein levels was also observed, from 64 g/L on the post-drainage assessment to 61 g/L on day 5, 59 g/L on day 8, and 58 g/L on day 18. Serum calcium remained mildly decreased throughout follow-up. Renal function remained preserved. Hematologically, hemoglobin levels remained overall stable, while the platelet count, initially 143 × 10³/mm³ after drainage, subsequently increased and remained within the normal range.

A lipid profile obtained on day 8 showed no notable abnormalities, with a total cholesterol level of 1.52 g/L, HDL cholesterol of 0.45 g/L, LDL cholesterol of 0.882 g/L, and triglycerides of 0.94 g/L.

Subsequent clinical course and patient outcome

Pleural drainage showed an overall decreasing trend during hospitalization and eventually ceased. As no further pleural fluid was collected, the chest tube was subsequently removed. No additional surgical intervention was considered necessary. Octreotide was discontinued after 13 days of treatment. The patient was subsequently discharged home without a chest tube, with a follow-up appointment scheduled with the thoracic surgery team.

Pleural drainage showed an overall decreasing trend during hospitalization and eventually ceased. As no further pleural fluid was collected, the chest tube was removed. No additional surgical intervention was required. Octreotide was discontinued on the same day, after 13 calendar days of treatment. The patient was subsequently discharged home without a chest tube. No detailed post-discharge follow-up information was available.

## Discussion

To place our case in the context of previously reported post-thymectomy chylothorax cases, its main clinical characteristics, management, and outcomes were compared with those reported by Zhang et al. [6], Kato et al. [7], and Kim et al. [8], as summarized in Table 5.

**Table 5:** Comparison of our case with previously reported cases of chylothorax following thymectomy. VATS, video-assisted thoracoscopic surgery; ED, emergency department; ICU, intensive care unit; POD, postoperative day; TPN, total parenteral nutrition; CT, computed tomography.

Characteristic	Our case	Zhang et al. (2018) [6]	Kato et al. (2023) [7]	Kim et al. (2016) [8]
Age/sex	50 years/male	57 years/male	46 years/female	50 years/male
Preoperative myasthenia gravis	Yes	No	No	No
Indication/pathology	Type B2 thymoma	Type AB thymoma	Type A thymoma	Anterior mediastinal mass
Masaoka stage	Stage I	Stage I	Stage I	Not specified
Surgical approach	VATS thymectomy	VATS extended thymectomy	Total thymectomy via median sternotomy	Total thymectomy via median sternotomy
Onset/diagnosis of chylothorax	Postoperative day 21	Postoperative day 3	Postoperative day 17	Postoperative day 2
Location	Left	Unilateral	Right	Bilateral
Clinical presentation	Acute respiratory distress with massive pleural effusion	Milky and turbid pleural effusion	Massive right pleural effusion	Change in bilateral chest tube drainage from clear to milky/turbid fluid
Notable drainage	1,500 mL initially in the ED; subsequently 4,000 mL in ICU	-POD3 (diagnosis): 400 mL/24h	Approximately 3,100 mL after drainage	470 mL/day at diagnosis → <150 mL/day during fasting → turned milky again after resuming normal diet (but still <150 mL/day) → 90 mL/day on low-fat diet
		-Fasting phase (POD3–9): stable at 300–350 mL/day		
		-After resuming water intake (POD9–15): increased to 600–700 mL/day		
		-After low-fat diet (from POD16): dropped from ~500 to 100 mL/day		
Nutritional management	Strict fat-free diet	Fasting + total parenteral nutrition from POD3 → reintroduction of oral water intake at POD9 → low-fat diet started at POD16	Fasting and total parenteral nutrition	Fasting + TPN from POD2 (1 week) → resumed normal diet → low-fat diet (3 days) → resumed normal diet
Octreotide	Yes	Not reported as part of treatment	Not reported as part of treatment	Not administered (deliberately avoided due to patient's diabetes; risk of hypo-/hyperglycemia)
Invasive treatment	None	Thoracic duct ligation proposed but refused	Lipiodol lymphangiography + pleurodesis; no surgical ligation	None
Outcome	Resolution of pleural drainage; octreotide discontinued after 13 days; discharged home.	Resolution with conservative treatment; chest tube removed on POD26; uneventful discharge; no clinical or radiographic evidence of recurrence at 3-month follow-up.	Resolved after lipiodol lymphangiography and OK-432 pleurodesis; chest tube removed the next day; discharged on POD27; no recurrence at 3 months.	Resolution with conservative treatment alone; chest tube removed the day after drainage dropped to 90 mL/day; discharged on POD16; no residual pleural effusion on CT at 3-month follow-up

Rarity and pathophysiological mechanism

Chylothorax is an uncommon complication following thymectomy. Huang et al. reported three cases among 217 patients undergoing extended thymectomy for myasthenia gravis, corresponding to an incidence of 1.38%. They suggested that, rather than involving an obvious injury to the main thoracic duct, postoperative chylous leakage may result from damage to small mediastinal lymphatic vessels or thoracic duct branches during extensive dissection of the perithymic fatty tissue. These lymphatic structures may not be readily identifiable during surgery [3]. Several mechanisms have been proposed to explain this complication. Kim et al. suggested that chylous leakage after thymectomy may result from injury to small lymphatic vessels within the anterior mediastinum rather than from direct damage to the main thoracic duct [8]. Similarly, Zhang et al. considered that the extensive removal of fatty tissue surrounding the thymus may expose small mediastinal lymphatic channels to inadvertent injury, thereby accounting for postoperative chylothorax despite the absence of an obvious thoracic duct lesion [6].

Timing of onset and clinical presentation

The timing of chylothorax onset represents a notable feature of our case. In our patient, the complication became clinically apparent on postoperative day 21, when he presented with acute respiratory distress associated with a massive left pleural effusion. This presentation was considerably later than in most previously reported cases. Kim et al. described bilateral chylothorax on postoperative day 2, identified by the appearance of milky and turbid fluid in the pleural drains [8]. Similarly, Zhang et al. reported chylothorax on postoperative day 3, with milky and turbid pleural drainage [6]. In contrast, Kato et al. described a delayed presentation on postoperative day 17, when a massive right pleural effusion was detected after no significant pleural effusion had been observed on postoperative day 7 [7].

More recently, Jaiswal et al. reported chylothorax following VATS extended thymectomy, detected as milky fluid in the intercostal drain on postoperative day 2. This further contrasts with the delayed onset observed in our patient despite the use of a minimally invasive surgical approach [9].

Taken together, these reports show that post-thymectomy chylothorax is most often recognized during the early postoperative period, whereas delayed presentations may also occur. The onset on postoperative day 21 in our patient therefore represents a particularly delayed presentation compared with the cases discussed above.

Diagnosis of chylothorax

The diagnosis of chylothorax relies primarily on pleural fluid analysis. Chylous fluid typically presents as a lymphocyte-predominant, milky exudate, although its appearance may occasionally be serous or blood-tinged, and a milky effusion alone is not specific enough to distinguish chylothorax from empyema or pseudochylothorax. Pleural fluid triglyceride levels above 1.24 mmol/L (110 mg/dL), together with cholesterol levels below 5.18 mmol/L (200 mg/dL), support the diagnosis. However, triglyceride levels may be below this threshold in approximately 15% of cases and can be influenced by the patient's nutritional status. Therefore, when clinical findings remain suggestive despite lower triglyceride levels, lipoprotein electrophoresis should be performed to detect chylomicrons and confirm the diagnosis [4].

In our patient, the diagnosis was biochemically confirmed by a pleural fluid triglyceride concentration of 188 mg/dL, exceeding the diagnostic threshold, and the detection of chylomicrons. These findings were consistent with the milky appearance of the drained fluid and the marked lymphocytic predominance (96%).

Drainage volume and consequences of chyle loss

Persistent chyle loss may lead to important nutritional and metabolic consequences. Chyle contains proteins, lipids, fat-soluble vitamins, electrolytes, lymphocytes, and immunoglobulins. Consequently, prolonged chylous drainage may contribute to nutritional deficiencies, electrolyte disturbances, and immunosuppression, highlighting the importance of monitoring nutritional and biological parameters during management [10].

In our patient, chyle output was particularly substantial at presentation, with 1.5 L drained in the emergency department followed by an additional 4 L after ICU admission. Pleural drainage remained high on the following day, reaching 1.1 L/24 h. Thereafter, drainage volumes fluctuated but showed an overall decreasing trend during hospitalization. During hospitalization, total serum protein levels decreased from 64 g/L at admission to 58 g/L by day 18, while hypokalemia was also observed.

The initial drainage volume in our patient was higher than that reported in previously published cases. Zhang et al. reported an initial chylous output of 400 mL/24 h on postoperative day 3, which subsequently varied according to nutritional intake, ranging from 300-350 mL/day during fasting to 600-700 mL/day after resumption of water intake [6]. Kim et al. similarly reported an initial output of 470 mL/day, which decreased to less than 150 mL/day during fasting and subsequently to 90 mL/day with a low-fat diet [8].

Conservative management: nutritional therapy and octreotide

Conservative management aims to limit lymphatic flow through the thoracic duct, thereby favoring spontaneous sealing of the leak. Dietary strategies typically involve restricting long-chain fat intake while providing medium-chain triglycerides, which are taken up directly by the portal venous system rather than through intestinal lymphatics, thus reducing chylous output. Total parenteral nutrition can be considered when chyle output is substantial or when dietary measures alone fail to achieve adequate control. Somatostatin and its synthetic analogue octreotide are also used as adjuncts in various forms of chylothorax, including postoperative cases, though consensus is lacking regarding the optimal regimen, dosage, and route of administration [10].

In our patient, a strict fat-free diet was initiated after recognition of the chylothorax. Octreotide was introduced on day 7, with a 100 µg slow intravenous loading dose followed by continuous infusion at 50 µg/h. Zhang et al. initially managed their patient with fasting and total parenteral nutrition from postoperative day 3. Oral water intake was reintroduced on postoperative day 9, followed by a low-fat diet from postoperative day 16. Octreotide was not administered in this case [6]. Kato et al. also adopted a conservative nutritional approach based on fasting and total parenteral nutrition. Octreotide was not reported as part of the treatment [7]. Kim et al. similarly initiated fasting and total parenteral nutrition on postoperative day 2. After one week, a normal diet was resumed; however, the pleural fluid became milky again, prompting a switch to a low-fat diet for three days before normal feeding was reintroduced. Octreotide was deliberately avoided because of the patient's diabetes and the potential risk of glycemic disturbances [8].

Overall, conservative management was the initial therapeutic approach across these cases, although nutritional strategies and the use of octreotide varied according to the clinical context.

Invasive management and indications for intervention

When conservative management fails, escalation to invasive treatment may be required. Proposed criteria for surgical intervention include persistent high-output chyle loss exceeding 1.5 L/day in adults, continued chest tube drainage for more than two weeks, or rapid deterioration in nutritional status. Earlier intervention may be considered in postoperative chylothorax. Thoracic duct ligation is the surgical technique most frequently reported in the literature, while lymphangiography with thoracic duct embolization provides a less invasive interventional alternative [10].

In our patient, no additional invasive intervention was required, as pleural drainage showed an overall decreasing trend and eventually ceased. The chest tube was subsequently removed, and octreotide was discontinued after 13 days of treatment. As no indication for surgical management was retained, the patient was discharged home.

Management beyond conservative therapy differed among the reported cases. In the case described by Zhang et al., thoracic duct ligation was proposed because of persistent chylous drainage but was declined by the patient, and conservative management was therefore continued until resolution [6]. In contrast, Kato et al. performed Lipiodol lymphography to localize the leakage site after the effusion became slightly milky again following resumption of oral feeding. The procedure was followed by a further reduction in pleural drainage, and chemical pleurodesis with OK-432 was subsequently performed as an additional precautionary measure [7].

## Conclusions

Postoperative chylothorax is an uncommon complication of thymectomy and may occasionally present in a delayed manner. This case describes a large left-sided chylothorax presenting with acute respiratory distress 21 days after VATS thymectomy, confirmed by pleural fluid triglyceride measurement and chylomicron detection. Despite the substantial initial chyle output, the patient achieved a favorable outcome with conservative management combining pleural drainage, dietary fat restriction, and octreotide, without the need for additional invasive or surgical intervention. Pleural drainage progressively decreased and eventually ceased, allowing chest tube removal, and the patient was discharged home. This case highlights the importance of considering chylothorax in patients presenting with pleural effusion even several weeks after thoracic surgery and of closely monitoring drainage output, nutritional status, and laboratory parameters during conservative management. However, the individual contribution of each therapeutic intervention to the favorable outcome cannot be determined.
